# Challenges and Achievements of Peptide Synthesis in Aqueous and Micellar Media

**DOI:** 10.1002/cbic.202500099

**Published:** 2025-05-21

**Authors:** Francesca Bordignon, Alessandro Scarso, Alessandro Angelini

**Affiliations:** ^1^ Dipartimento di Scienze Molecolari e Nanosistemi Università Ca’ Foscari Venezia via Torino 155 30172 Mestre Venezia Italy; ^2^ European Centre for Living Technology (ECLT) Ca’ Bottacin Dorsoduro 3911, Calle Crosera 30123 Venezia Italy

**Keywords:** amide bonds, aqueous medium, micellar media, peptide, peptide bonds, solid‐phase peptide syntheses, water

## Abstract

Peptides are being increasingly explored for drug development as well as other applications, ranging from research tools to food additives. This growing interest in peptides has led to the need to develop new sustainable synthetic approaches for this class of molecules. The present review article focuses on the synthesis of peptides in aqueous media to drastically reduce organic solvent use and its consequent environmental impact. After some pioneering investigations about solid‐phase peptide synthesis in water, the field is experiencing a renaissance also for the synthesis in solution spurred by increasing applications enabled by micellar catalysis. In this contribution, the challenges and opportunities offered by using aqueous and micellar media in the chemical synthesis of peptides are critically discussed.

## Graphical abstract

Peptide synthesis either on solid support or in solution for the potential development of new peptide drugs is facing a new challenge consisting in the progressive elimination of toxic polar aprotic solvents in favor of the more environmentally friendly water. The present review underlines challenges, opportunities, and achievements in this emerging field of research.

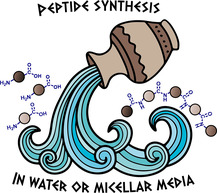

## 1 Introduction

Peptides are a unique class of biomolecules composed of a series of well‐ordered amino acids, typically ranging from 2 to 50 residues, that play fundamental roles in human physiology.^[^ ^1^ ^]^ Their intrinsic properties, coupled with the advent of new peptide discovery technologies, improved synthetic methods and delivery strategies, have led to a continuously growing number of peptide‐based drugs that are currently used in a wide range of therapeutic areas. A total of 33 non‐insulin peptide drugs have been approved worldwide since 2000. Overall, ≈50–100 peptides are available on the market as active pharmaceutical ingredients (APIs),^[^ ^2^ ^]^ with hundreds in clinical trials and many more under preclinical development stages.^[^ ^3^ ^]^ Overall, peptides account for about 10% of the market of API,^[^ ^4^ ^]^ and their relevance is constantly increasing. In addition to pharmaceutical applications, peptides are increasingly used as research tools and food additives.

Nowadays peptides can be produced using both biological and chemical methods. The latter approach is preferred when specific structural modifications are required, such as the incorporation of non‐natural amino acids or the functionalization with non‐peptide units. Small scale chemical synthesis of peptides often relies on the classical solid‐phase peptide synthesis (SPPS) approach developed by Merrifield in 1963.^[^ ^5^ ^]^ This methodology is based on the use of polymeric resin solid supports to which the *C*‐terminal amino acid is chemically linked, followed by a sequence of *N*‐deprotection and coupling with the *C*‐activated and *N*‐protected incoming amino acid. Frequently, side chain protection is requested as well. The synthesis of the peptide occurs on the solid support, and by applying solvent washes, excess of reagents and coupling side products can be removed, greatly simplifying the subsequent purification steps. The latter are usually needed to remove truncated peptide sequences and/or peptide sequences that have undergone side reactions such as epimerization and other chemical modifications (e.g., oxidation, deamidation, dehydration, etc.) that may affect the final peptide sequence, structure, and biological activity. SPPS on solid support is usually carried out in *N*,*N*‐dimethylformamide (DMF) as solvent. However, a recent study using Oxyma Pure/*tert*‐butyl‐ethyl carbodiimide protocol in *N*‐butyl pyrrolidone/dimethyl carbonate showed that it is possible to synthesize peptide sequences using unprotected amino acids such as arginine (Arg), histidine (His), tryptophan (Trp), and tyrosine (Tyr).^[^ ^6^ ^]^

For peptides characterized by the presence of difficult coupling steps, the synthesis on solid support is not optimal and a switch to a more classical synthesis in solution is unavoidable (liquid‐phase peptide synthesis, LPPS). In the latter case, the experimental conditions can be tailored more precisely to ensure high yields, even though the purification steps become tedious.

Looking to peptide synthesis from another perspective, the formation of amide bonds is a very well‐known reaction. Indeed, in the chemical synthesis of drugs and their intermediates, the formation of amides is one of the most common reactions.^[^ ^7^ ^]^ The advent of green chemistry^[^ ^8^, ^9^ ^]^ and a growing environmental awareness are driving a shift towards the implementation of sustainable approaches by companies.^[^ ^10^, ^11^ ^]^ In particular, the main issue is due to the high waste production that the synthesis of complex and multistep targets can cause. The current state‐of‐the‐art technologies for peptide couplings are characterized by general waste amounts on the order of 3000−15000 kg per kg of API.^[^ ^12^ ^]^ It is important to notice that solvents account for the largest amount of waste generated,^[^ ^13^, ^14^ ^]^ since their recycling is costly and energy consuming, contributing negatively to green metrics.^[^ ^15^, ^16^ ^]^ Over the past 10–15 years, pharmaceutical companies have released a number of solvent selection guides to address the issue of minimizing solvent waste and replacing them with safer alternatives.^[^ ^17^, ^18^ ^]^ Nowadays, among the solvents generally used for peptide synthesis, DMF, *N*‐methyl‐2‐pyrrolidone (NMP) and dichloromethane (DCM) remain the most preferred ones, even though their toxicity and concerns are well‐known especially in Europe and the United States.^[^ ^19^ ^]^ Although less toxic alternative solvents have been proposed,^[^ ^20^ ^]^ they have so far found limited applications, mainly for economic reasons.

An emerging strategy that is attracting much interest, with examples of possible extension into pharmaceutical synthesis, is the use of water as a reaction medium,^[^ ^21^, ^22^, ^23^ ^]^ where the addition of specific surfactants provides a solution for the solubilization or dispersion of organic apolar substrates.^[^ ^24^, ^25^, ^26^, ^27^ ^]^ Due to their amphiphilic nature, surfactants aggregate in water to form micellar nanoenvironments in solution, where reagents and catalysts combine, resulting in improved reactivity and selectivity. Nowadays, many chemical transformations are possible using aqueous media with surfactants. In this context, a great contribution is due to the designer surfactants introduced by Lipshutz^[^ ^28^, ^29^, ^30^ ^]^ such as TPGS‐750‐M,^[^ ^31^ ^]^ PS‐750‐M,^[^ ^32^, ^33^ ^]^ and the polymeric additive hydroxypropyl methylcellulose (HPMC).^[^ ^34^ ^]^ All these surfactants are currently commercially available and find application in numerous chemical reactions with beneficial effects in terms of milder experimental conditions, higher rates and improved selectivity.^[^ ^35^, ^36^, ^37^ ^]^

**Figure 1:**
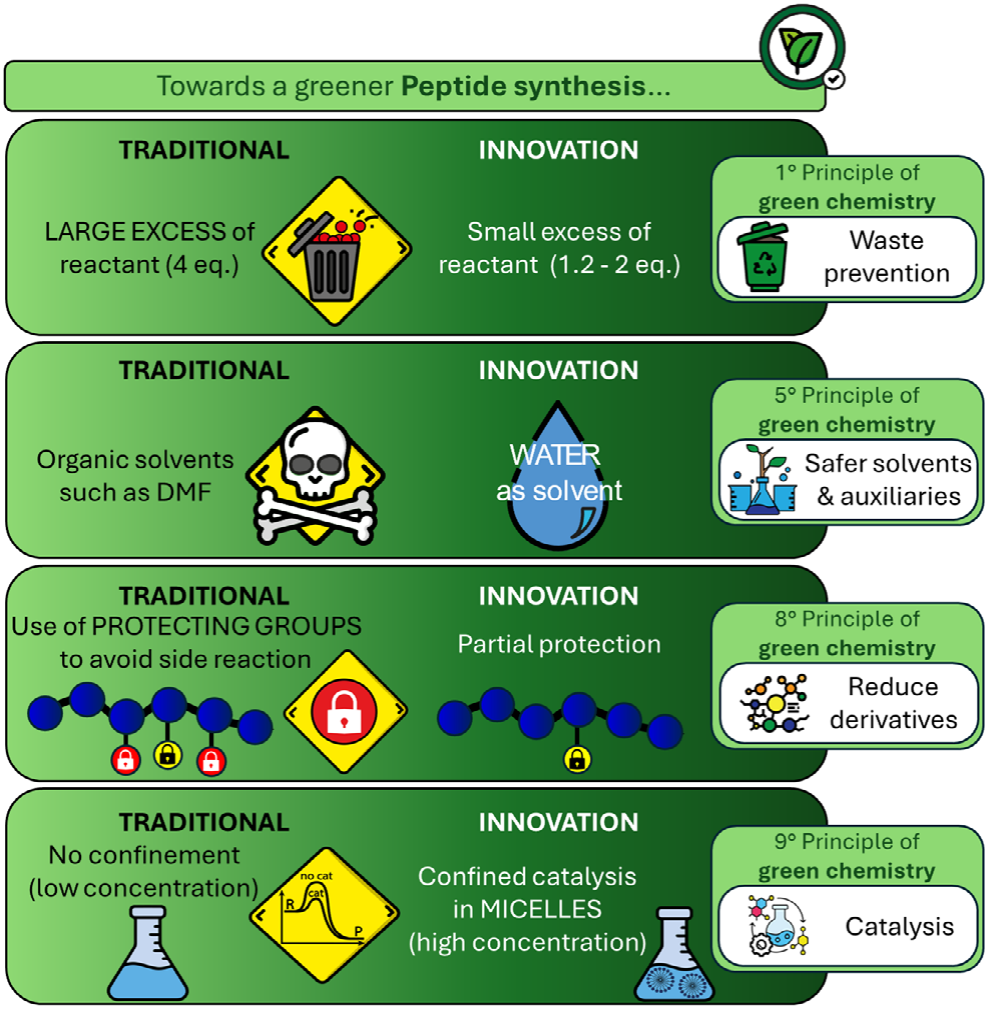
Overview of potential improvements needed to shift peptide synthesis from traditional organic solvents to aqueous media.

The two emerging trends, the development of peptide therapeutics and the use of aqueous media for drug synthesis, are increasingly showing points of contact in recent years. In this review, we intend to provide a critical overview of the state‐of‐the‐art and the challenges and strategies proposed so far by the scientific community to synthesize peptides in aqueous media to achieve many improvements in terms of green chemistry practice (**Figure** **1**). The intrinsic issues that must be overcome are related to the insufficient solubility in water of the molecules involved and to the possible side reactions due to the acidic and nucleophilic nature of water. The use of additives provides valuable starting solutions that deserve further efforts to be widely available. The review focuses initially on the discussion of general aspects of peptide bond formation in water, followed by a series of examples of SPPS and LPPS performed in aqueous media both with and without the aid of surfactants. Future perspectives in the field are discussed in the conclusion and outlook section, highlighting potential new directions for this emerging field of research.

## 2 Amide and Peptide Bond Formation in Aqueous Media

Amide coupling in process chemistry for pharmaceutical synthesis is a well‐established reaction, on which a large body of literature already exists.^[^ ^38^ ^]^ A care is often placed on the coupling system and the general experimental conditions, with particular attention when dealing with the formation of peptide bonds. Regarding the choice of solvent, many solvents are compatible with different activated acid derivatives. For instance, acyl chlorides can provide amides by reaction with amines even in water under Shotten Baumann conditions, as well as aided by the presence of surfactants,^[^ ^39^, ^40^ ^]^ with the polar protic solvent favoring the reaction without being detrimental for the acyl chloride. Thus, the choice of solvent is mainly a matter of solubility and compatibility and cost and ease of isolation of the amide, as well as ease of removal of the solvent from the final product. Among possible solvents, tetrahydrofuran (THF), DCM, and DMF are the most common solvents employed, while water still represents a possible choice in many cases.^[^ ^41^ ^]^ Indeed, a survey of the literature about pharmaceutical companies showed that water is the third most frequently used solvent for amide bond formation.^[^ ^36^ ^]^

**Figure 2:**
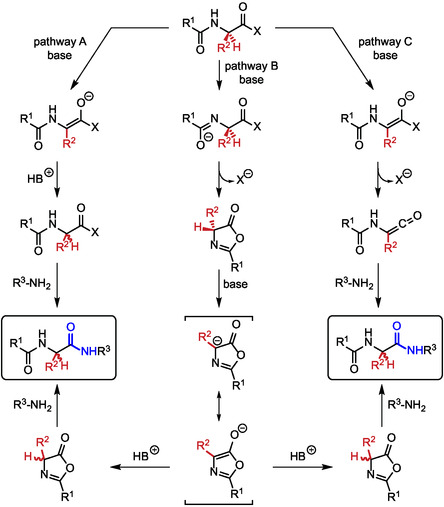
Plausible racemization pathways during peptide bond formation as a consequence of activation of the carboxylic acid moiety.

The scenario changes dramatically when it comes to amino acid couplings and peptide bonds. This is due primarily to the enhanced solubility problems for apolar substrates and the influence of solvents on possible racemization side effects. Indeed, the activation of amino acids could lead to the formation of intermediate oxazolone species (also known as azlactone) which in the presence of bases could lose the hydrogen atom on the stereocenter leading to racemization upon protonation (**Figure** **2**). Although racemization is the result of the combination of many factors, the polarity of the solvent can also play a role. It is known that more polar solvents favor this side reaction, with acetonitrile and THF being preferred over DMF on this specific issue (Figure 2).^[^ ^42^ ^]^ Furthermore, the need to use bulky and hydrophobic protecting groups in peptide synthesis makes amino acids highly apolar, thus limiting their solubility in media such as water, hindering peptide bond formation and ultimately reducing the yields.

**Figure 3:**
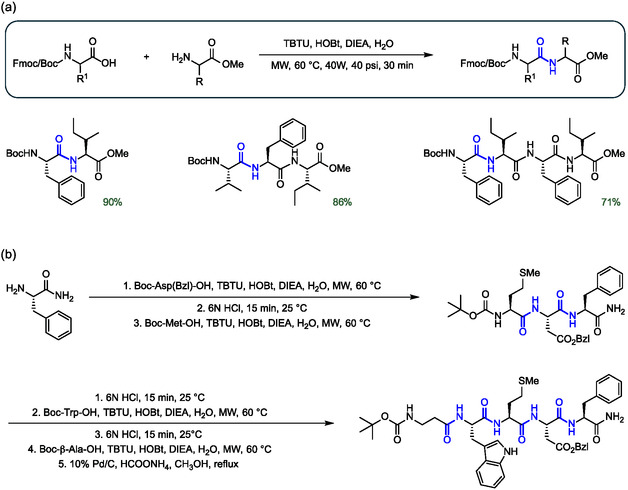
Examples of peptide synthesis with TBTU as condensing agent in aqueous media under microwave irradiation. a) General reaction scheme with representative examples of peptide bond formation. b) Application to the multistep synthesis of the bioactive pentapeptide pentagastrin. The peptide bonds formed are highlighted in blue.

The use of microwave irradiation in peptide synthesis to promote rapid amide couplings has been introduced several years ago. This technique can be very advantageous for peptide synthesis in water.^[^ ^43^ ^]^ The combination of TBTU/HOBt/DIEA as coupling system under microwave irradiation at 60 °C allowed the synthesis of several peptides with a reaction time of 30 min in pure water (**Figure** **3**a). Both Boc‐ and Fmoc‐protected amino acids were used efficiently, with generally better yields for the former. The method was upscaled to nearly 1 g without complications, with the added advantage of using only 1.2 equivalents of the coupling amino acid, a much lower excess than typically used in organic media. The approach was also extended to the multistep synthesis of the bioactive pentapeptide pentagastrin. The peptide was obtained in an overall yield of 42% by alternating the coupling in aqueous media under microwave irradiation conditions with Boc deprotection in the presence of 6 m HCl for 15 min (Figure 3b).

## 3 SPPS in Aqueous Media

SPPS is a well‐established method for the synthesis of medium‐length peptide sequences.^[^ ^44^ ^]^ The methodology was developed by Merrifield and is based on the attachment of the *C*‐terminal amino acid to a polymeric resin solid support, followed by an amide coupling sequence with the second amino acid carrying Boc and, more frequently, Fmoc as the protecting group of the α‐amino function.^[^ ^5^ ^]^ The heterogenization of the peptide favors rapid washing after each coupling offering many advantages in terms of simplicity and speed. Subsequent deprotection of the *N*‐terminal group and coupling with a new incoming amino acid can be repeated multiple times, allowing automation. SPPS is currently an extremely robust approach, with instrumentation allowing automated peptide preparation in shorter times.

**Figure 4:**
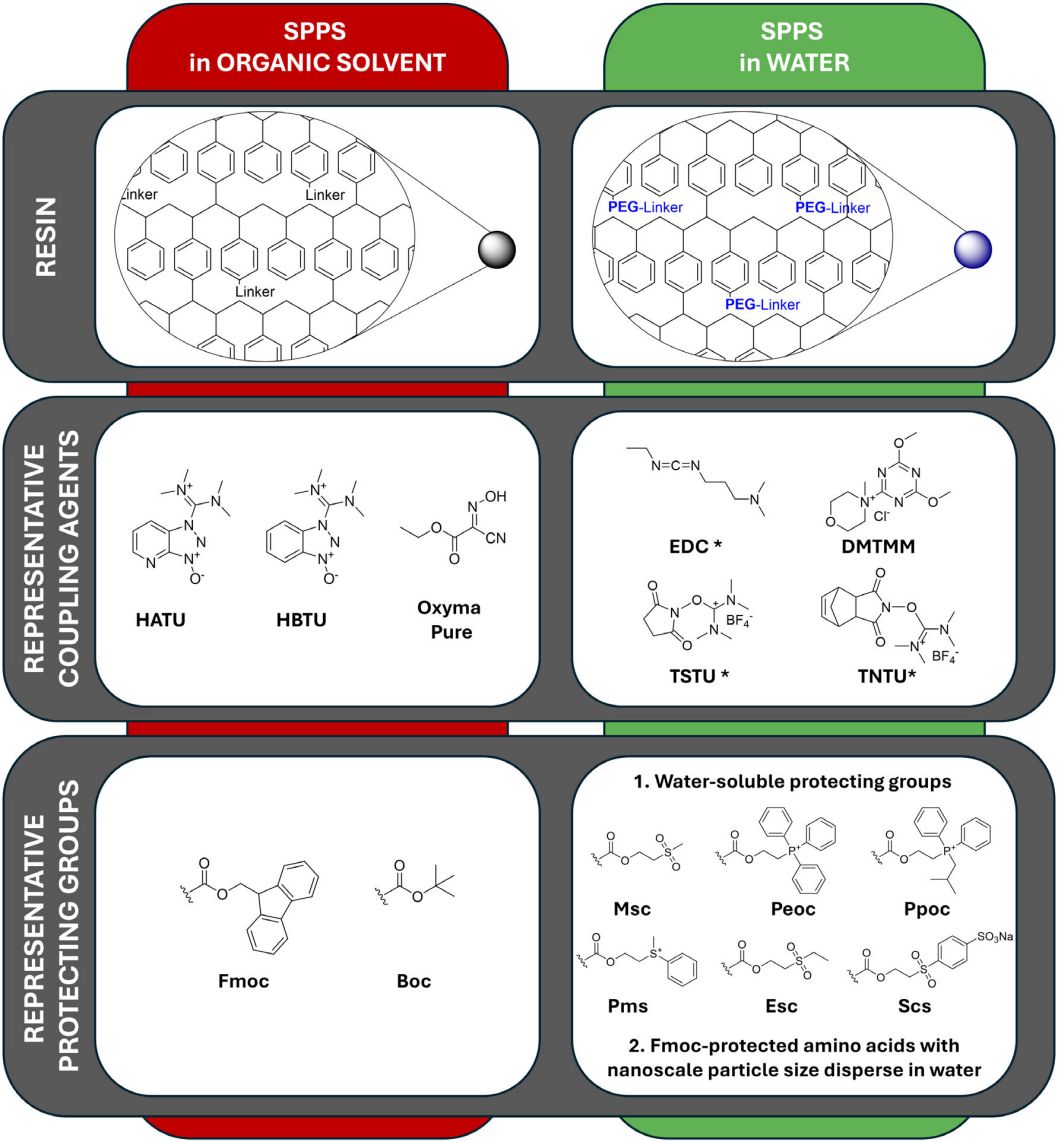
Comparison between traditional SPPS in organic solvents (left) and the alternative approach in aqueous media (right), showing the molecular differences requested in terms of polymeric resin, coupling agents and protecting groups (* = sometimes used with additives).

Despite its great success, this technique requires extensive use of polar aprotic solvents, particularly DMF, resulting in the production of large amounts of waste organic solvents. Over the years, several alternative organic solvents have been proposed for peptide synthesis.[^20^, ^45^] These include ethyl acetate, 2‐methyltetrahydrofurane, alcohols, and others characterized by various advantages in terms of green chemistry principles. Albeit being the solvent of choice in nature and a green solvent *par excellence*, the use of water as medium has for many years been not a straightforward solution, particularly because of solubility issues. The investigation of SPPS in water requires a change of perspective considering that the hydrophobic reagents commonly used for coupling and deprotection on SPPS must maintain good solubility in water. Furthermore, the polymeric resin must also exhibit a good degree of swelling in water to ensure efficient diffusion of the reagents to react with the growing peptide chains. This last problem has been overcome with the advent of resins consisting of copolymers with a low crosslinking polystyrene matrix, grafted or based entirely on hydrophilic polyethylene glycol (PEG; **Figure** **4**). Although the introduction of PEGylated resin has enabled efficient swelling in water and other solvents, the low aqueous solubility of amino acids protected with bulky hydrophobic Boc or Fmoc protecting groups still represents a critical limitation for efficient SPPS synthesis. In this context, two opposing solutions have been proposed. One is based on the use of water‐soluble amino acids, while the second is based on the use of water‐insoluble amino acids. Here we report the main features of the two approaches, while for the details we refer to a recent review.^[^ ^46^ ^]^

### 3.1 SPPS with Water‐Soluble Amino Acids

The first approach relies on protecting groups for the amino functional group that are intrinsically soluble in water. Due to the presence of charged heteroatoms, these groups ensure good solubility in water through ion–dipole interactions and hydrogen bonds. Representative examples of protecting groups for the amino group, with their respective advantages and disadvantages, are summarized in **Table** **1**.

**Table 1:** Examples of water‐solubilizing protecting groups for amino acids for SPPS in aqueous media.

Ref.	Protecting group	Deprotection	Advantages	Disadvantages
[70]	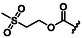 **Msc**	4 N NaOH Mixture dioxane‐methanol (0.25:7.5:2.25)	High acid stability Removable in homogeneous phase giving a volatile ether	Not detectable by optical absorption spectroscopy
[71]	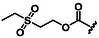 **Esc**	0.025 m NaOH (3 × 3 min) or 2.5% Na_2_CO_3_ (3 × 5 min) in 50% aqueous EtOH	May be used also in organic solvents	Quantitative removal in water is slow Difficult optical detection due to the lack of an intense chromophore
[72, 73]	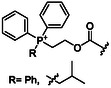 **Peoc, Ppoc**	0.1 N NaOH in methanol/water (9:1) 1 min	It is possible to modulate elimination rate by changing substituent on P	Highly sensitive to bases
[74]	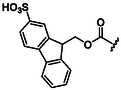 **Sulfmoc**	Anhydrous morpholine or aqueous 1% Et_3_N, 1% Na_2_CO_3_,0.1 N NH_4_OH, or 0.1 N NaOH	**–**	**–**
[75]	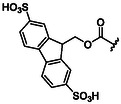 **Smoc**	0.2 m NaOH, Ethylenediamine 10% v/v, Piperazine 5% w/v, Ammonia 10% v/v, Piperidine 20% v/v in aqueous solution for 5 min	Pronounced fluorescence allows for real‐time monitoring	‐
[76]	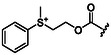 **Pms**	5% NaHCO_3_ (1 or 2 × 30 min) and 0.01 m NaOH (2 × 3 min) aqueous solution	High base lability	Rather unstable
[77]	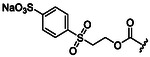 **Sps**	5% Na_2_CO_3_ (5 min) or 0.025 m NaOH (2 × 3 min) aqueous solution	Stable solids Detectable by UV	**–**

### 3.2 SPPS in Aqueous Media with Water‐Insoluble Amino Acids

**Figure 5:**
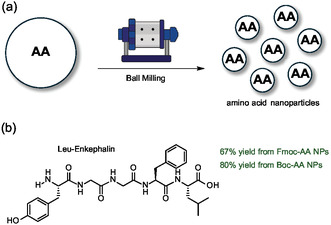
a) Treatment with ball milling provides amino acid nanoparticles suitable for direct SPPS in water. b) Synthesis with Fmoc‐ and Boc‐protected amino acid nanoparticles of Leu‐Enkephalin peptide (H‐Tyr‐Gly‐Gly‐Phe‐Leu‐OH). AA: amino acid.

An alternative approach that does not require the use of specific water‐soluble protecting groups for the amino acids has been proposed by Hojo and colleagues. The strategy consists in using Fmoc‐protected amino acids with nanoscale particle sizes that make them highly dispersible in aqueous solution (**Figure** **5**a).^[^ ^47^ ^]^ The smaller the particle size, the greater the surface area and reactivity in water. The Fmoc‐protected amino acids are then subjected to a ball milling process obtaining particles of about 700 nm characterized by a high surface area forming nanosuspensions that can be used with resin in water for SPPS.

The effectiveness of this approach was demonstrated in the coupling of an amino acid in the form of nanoparticles with an amino acid grafted onto a resin, observing a quantitative yield after 30 min. This method greatly simplifies the purification step, since excess reagents can be easily removed by centrifugation. Moreover, standard Fmoc‐protected amino acids can be used and Fmoc‐protected amino acid nanoparticles have been shown to be effective for the synthesis of the pentapeptide Leu‐enkephalin (Figure 5b). The yields obtained with Fmoc‐protected amino acid nanoparticles were higher than those of traditional Fmoc‐protected amino acids. Moreover, the addition of a surfactant in solution further increased this discrepancy. More recently, the same authors extended the approach to Boc‐protected amino acids processed into nanoparticles for SPPS by means of microwave irradiation.^[^ ^48^ ^]^

Using PEG‐grafted resin and Boc‐protected amino acid nanoparticles dispersed in water in a 0.2% Triton X‐100 solution, the authors observed complete couplings within 1 min at 70 °C using 4‐(4,6‐dimethoxy‐1,3,5‐triazin‐2‐yl)‐4‐methylmorpholinium chloride (DMTMM) as the coupling agent. The method has been successfully applied to the synthesis of numerous peptides from 2 to 5 amino acids. In particular, the authors performed the synthesis of Leu‐enkephalin with yields above 80% and 90% purity (Figure 5b). Furthermore, the authors demonstrated that the method is also suitable for the synthesis of Val‐Ala‐Val‐Ala‐Val‐Gly‐OH, a 6‐amino acids peptide known for its tendency to aggregate, without presenting such issues.

**Figure 6:**
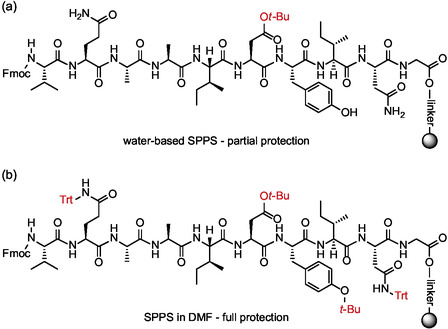
The use of water as reaction medium a) enables the efficient SPPS synthesis of ACP peptide (H‐Val‐Gln‐Ala‐Ala‐Ile‐Asp‐Tyr‐Ile‐Asn‐Gly‐OH) by avoiding the protection of amino acid side chains comprising phenol and amide groups, which is otherwise necessary when using traditional SPPS in DMF b).

Another key step towards a more sustainable peptide synthesis would undoubtedly be the possibility of avoiding the use of side chain protecting groups. Although the presence of these groups could prevent side reactions, their absence would greatly increase the solubility of amino acids in water, while reducing the cost of their production and the amount of organic waste generated during their synthesis. A step forward in this direction has recently been made and involves the use of a synthesis protocol that avoids the protection of the hydroxyl or amide side groups.^[^ ^49^ ^]^ The presence of an unprotected hydroxyl group may indeed lead to *O*‐acylation during the reaction with the incoming activated amino acid. By performing the reaction in an aqueous solution, the authors claimed that the formation of hydrogen bonds with water significantly reduced the nucleophilicity of this functional group. Coupling reactions were performed with all three amino acids containing a hydroxyl group (Ser, Thr, Tyr), at pHs ranging from 6 to 7 and using one equivalent of *N*‐methyl morpholine (NMM) as base. In none of the cases was any formation of *O*‐acylation products observed. Furthermore, the formation of hydrogen bonds between water and the unprotected hydroxyl group of the side chains can reduce the aggregation phenomena that would otherwise occur if the side chains were protected by bulky hydrophobic groups. For this reason, the synthesis of the acyl carrier protein (ACP) 10‐amino acids (65–74) peptide (**Figure** **6**) was accomplished by also removing the Asn and Gln protecting groups, obtaining the final product with a 26% yield.

Sometimes, for more difficult sequences, coupling reactions are conducted at higher temperatures to force the reaction to completion. To reach such temperature, microwave irradiation is often used. An interesting work by Galanis and colleagues investigated the use of microwave for the SPPS using water as medium, evaluating the use of amino acids with different protecting groups in combination with several classes of polymeric resins.^[^ ^50^ ^]^ Thanks to microwave irradiation, optimized coupling steps were performed in water at 75 °C for only 7 min. Using microwave‐assisted SPPS, the pentapeptide Leu‐enkephalin was successfully synthesized using both traditional Boc‐ and Fmoc‐protected amino acids. The coupling steps were shown to be efficient using both protection systems, ultimately leading to high peptide yields and no epimerization. Furthermore, the synthesis efficiency was improved by adding small amounts of a neutral surfactant (0.5% Triton‐X100).

A critical point in SPPS is the minimization of side reactions. This is especially essential for peptides used in pharmaceutical applications where the purity standards required are extremely high. One of the most well‐known side reactions that can occur in peptide synthesis is varying levels of epimerization at one or more sites.^[^ ^51^ ^]^ Indeed, changing the configuration of just one amino acid in a sequence can have dramatic effects on the biological properties of the peptide. Serious racemization problems can be encountered especially in the synthesis of peptide sequences containing His residues. In the case of His, this can occur following the abstraction of an α‐proton by the imidazole ring. In particular on this point, it has been shown that the use of a weaker base such as NMM along with a better control of pH conditions obtained by performing the synthesis in an aqueous environment rather than in organic media is useful in lowering the level of racemization.^[^ ^52^ ^]^ By exploiting microwave‐assisted SPPS in water with Fmoc‐amino acid nanoparticles, the authors successfully synthesized NPW30, a His‐containing hexapeptide, in high yield and with significantly reduced His racemization (D/L ratio < 3.3%).

**Figure 7:**
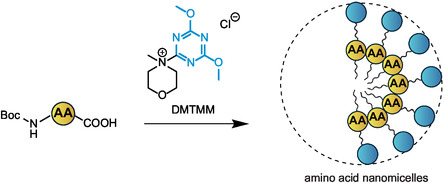
Reaction of Boc‐protected amino acids with DMTMM provides nanomicelles suitable for SPPS in water. The chemical moiety that confers water solubility is highlighted in cyan. AA: amino acid.

As an alternative to Fmoc‐protected amino acid nanoparticles, Hojo and colleagues have also proposed a nanomicellar approach to enhance the solubilization of protected amino acids in water by reaction with DMTMM as a coupling agent.^[^ ^53^ ^]^ The reaction provides intermediate amphiphilic species that self‐assemble forming aggregates in water with average mean diameter of 700 nm (**Figure** **7**).^[^ ^48^ ^]^ Such innovative approach was successfully used for the synthesis of the pentapeptide Leu‐enkephalin. By performing SPPS on HMBA‐PEG grafted resin and applying coupling reaction times of 3 min, the final peptide was obtained with an overall purity comparable to that obtainable using classical SPPS in the presence of organic solvents. This approach, which exploits the formation of nanomicelles in water and does not require protected nanoparticle‐sized amino acids, deserves further attention and represents a point of contact with peptide synthesis in aqueous solution with surfactants.

**Figure 8:**
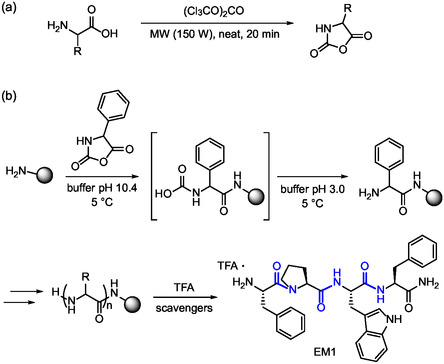
a) Synthesis of NCA amino acids that present activation of the carboxylic moiety and concomitant protection of the amino group. b) Application of NCA method for the synthesis of EM1 peptide (H‐Tyr‐Pro‐Trp‐Phe‐NH_2_). The peptide bonds formed are highlighted in blue.

A further reduction of waste generation has been proposed by Gentilucci and coworkers and involves the use of *N*‐carboxyanhydride (NCA) derivatives as activated amino acids. These are usually prepared under solvent‐free conditions by treatment of the amino acid with triphosgene under microwave irradiation and further reacted with PEG‐containing resins for SPPS in water. The advantage of NCAs is their mixed anhydride activator group, which also serves as a protecting amino group, easily removed after coupling (**Figure** **8**a).^[^ ^54^ ^]^ Overall, apart from amino acids, the only bulk chemical group was triphosgene with very good overall atom economy and waste reduction. The authors demonstrated the potential of this method by synthesizing the endogenous opioid peptide endomorphin‐1 (EM1) which was obtained in excellent yield and purity prior to semi‐preparative HPLC (Figure 8b).

## 4 LPSS in Aqueous Media with Surfactants and Additives

**Figure 9:**
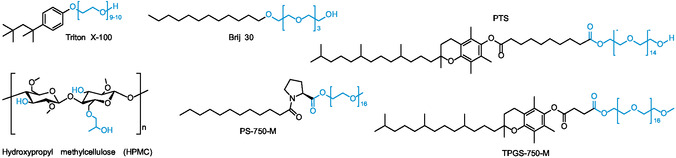
Molecular structure of the most common traditional and designer neutral surfactants and water additives. Hydrophobic and hydrophilic portions are indicated in black and cyan, respectively.

The addition of surfactants and additives of various nature in water leads to the formation of aggregates with different sizes and shapes (**Figure** **9**). These aggregates are characterized by internal hydrophobic spaces that are sheltered from bulk water by the hydrophilic portion of the amphiphiles. Such non‐polar nanoenvironments have been exploited for the development of catalysis in which apolar substrates come into contact thanks to the environment created by amphiphilic molecules. The high local concentration achieved is responsible for the overall accelerations observed when operating under micellar conditions compared to organic solvents. Additionally, surfactants with specific structures, such as TPGS‐750‐M^[^ ^28^ ^]^ and PS‐750‐M,^[^ ^55^ ^]^ may offer further benefits to spur reactivity and catalysis in water.

Given the importance of developing a robust and green amide bond synthesis, several contributions regarding the formation of peptide bonds in water aided by the addition of surfactants have already been reported in the literature. To achieve this goal, new and better performing designer surfactants have recently been described. In addition, the use of water additives such as hydroxypropyl methylcellulose (HMPC, Figure 9) has also been proposed to promote peptide binding in water. In the following section, we have critically discussed the most relevant recent contributions based on the chemical structure of the amphiphile employed. We have reported them in chronological order to highlight the progress made in terms of activity, selectivity and reduction of reaction times.

### 4.1 Surfactant TPGS‐750‐M

**Figure 10:**
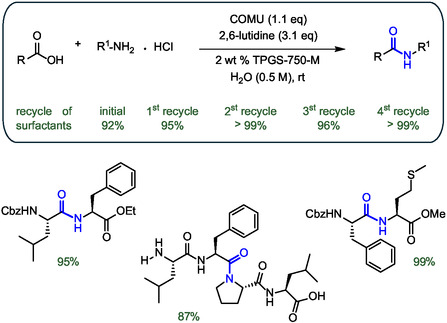
Synthesis of peptides in water in the presence of surfactant TPGS‐750‐M mediated by COMU as coupling agent. The peptide bond formed is highlighted in blue.

Lipshutz and colleagues began studying the potential of TPGS‐750‐M in peptide bond formation almost 10 years ago. TPGS‐750‐M is one of the most effective surfactants and provides spherical and worm‐like micelles in water with an average size of 50–60 nm. These micelles were tested as nanoenvironments for peptide bond formation in water using the highly active Oxyma‐derived uronium salt (1‐cyano‐2‐ethoxy‐2‐oxoethylidenaminooxy)‐dimethylaminomorpholino carbenium hexafluorophosphate (COMU) as a coupling agent between Boc‐, Fmoc‐, and Cbz‐protected amino acids with ester‐protected partners using 2,6‐lutidine as a base. This approach was efficiently applied for the synthesis of 19 different dipeptides and tripeptides, achieving yields above 74% with reaction times ranging from 1 to 4 h (**Figure** **10**).^[^ ^56^ ^]^ It is worth noting that the method proved to be efficient even for sterically hindered amino acids such as the *N*‐Cbz protected α‐aminoisobutyric acid (Z‐Aib‐OH). At the end of the reaction, the product was isolated by extraction with a minimal volume of organic solvent (e.g., *i*‐PrOAc), while the high‐water solubility of the urea and oxime by‐products of COMU ensured their removal with the aqueous waste. It was further demonstrated that the reaction medium containing the surfactant could be easily recycled at least four times with almost unchanged yields, leading to a procedure with an E factor (total mass of waste per unit mass of product) of 7.8, including also the aqueous waste.

**Figure 11:**
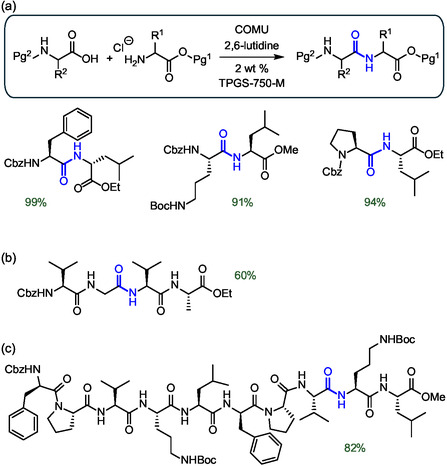
Synthesis of a) dipeptides, b) a tetrapeptide precursor of the anti‐microbial dermaseptin and c) a decapeptide gramicidin S in water with TPGS‐750‐M by *N*‐deprotection with H_2_ Pd/C followed by coupling with COMU. The peptide bond formed is highlighted in blue.

The robustness of the previous in‐water synthesis protocol with TPGS‐750‐M led Lipshutz's group to take a step forward by developing a deprotection/coupling sequence for the solution‐phase synthesis of peptides containing up to ten amino acids (**Figure** **11**).^[^ ^57^ ^]^ A key point for the success of the approach was the use of highly hydrophobic Cbz‐protected amino acids. This property was shown to favor their localization in micelles for efficient coupling. Furthermore, the Cbz‐protected amino acids could be easily deprotected by hydrogenation on 10% heterogeneous Pd/C catalyst without side products. Initially, a series of dipeptides were prepared by deprotection of the *N*‐terminal amino acid by hydrogenation, followed by degassing and replacement with an inert atmosphere before subsequent coupling with COMU. In some cases, a small addition of a cosolvent such as THF was required to ensure good mixing and to achieve high yields. It is worth noting that reactions involving both Tyr and Ser residues were performed without protecting groups on the side chain hydroxyl groups (Figure 11a). A series of longer peptides from tripeptides up to decapeptidesa were also prepared by Cbz deprotection of a dipeptide followed by coupling with the residual fragment. It is worth mentioning the peptide Cbz‐Val‐Gly‐Val‐Ala‐OEt, a precursor of the antimicrobial dermaseptin, which was obtained in two steps starting from a tandem [2 + 2] sequence with a final yield of 60% (Figure 11b). Similarly, the decapeptide Cbz‐D‐Phe‐Pro‐Val‐Orn(Boc)‐Leu‐D‐Phe‐Pro‐Val‐Orn(Boc)‐Leu‐OMe, a linear precursor of the antibiotic gramicidin S, was obtained via a convergent [8 + 2] approach with a final yield of 82% (Figure 11c). Overall, the synthetic protocol proved to be robust and reliable, in particular ensuring high stereochemical integrity of the amino acids, with virtually no evidence of epimerization. From a green chemistry perspective, the method was characterized by E factor values in the range of 10–15, depending on whether aqueous waste is included in the calculation. These values are one to two orders of magnitude lower than those typically reported for the synthesis of pharmaceutical molecules.

**Figure 12:**
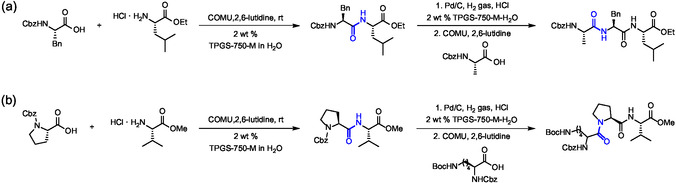
Synthesis of tripeptides a) Cbz‐L‐Ala‐L‐Phe‐L‐Leu‐OEt and b) Cbz‐L‐Lys(Boc)‐L‐Pro‐L‐Val‐OMe by sequential coupling, deprotection, and subsequent coupling in water with TPGS‐750‐M. The peptide bond formed is highlighted in blue.

Peptide bond formation in water with TPGS‐750‐M mediated by COMU and 2,6‐lutidine was further optimized for the step‐by‐step synthesis of two tripeptides.^[^ ^58^ ^]^ For this purpose, the authors used a protocol that required high concentration of substrates (typically 0.5 m), 3–4 h incubation for a simple coupling and 8 h for a two‐step deprotection/coupling process (**Figure** **12**). Optimization of the workup led to isolation of the pure product by simple extraction with minimal amounts of organic solvent like methyl *t*‐butyl ether (MTBE) or EtOAc and acidic/basic aqueous washings. The reactions were upscaled to grams with overall low E factors. Among the drawbacks of the protocol, couplings involving Gly residues were characterized by lower yields due to its lower lipophilicity and consequent lower affinity for the apolar core of micellar aggregates. Special care was taken to ensure efficient stirring of the reaction mixture, noting that yields were generally higher if most of the volume of the flask was occupied by the reaction mixture. For the same purpose, in some cases it was necessary to add up to 10% THF to ensure good mixing and good overall yields.

The use of TPGS‐750‐M has led to improvements in many examples of peptide synthesis in water, including cases where pivaloyl chloride was used as a reagent to first yield the mixed anhydride and then react with the free amino group of the incoming amino acid.^[^ ^59^ ^]^

### 4.2 Surfactant MC‐1

**Figure 13:**
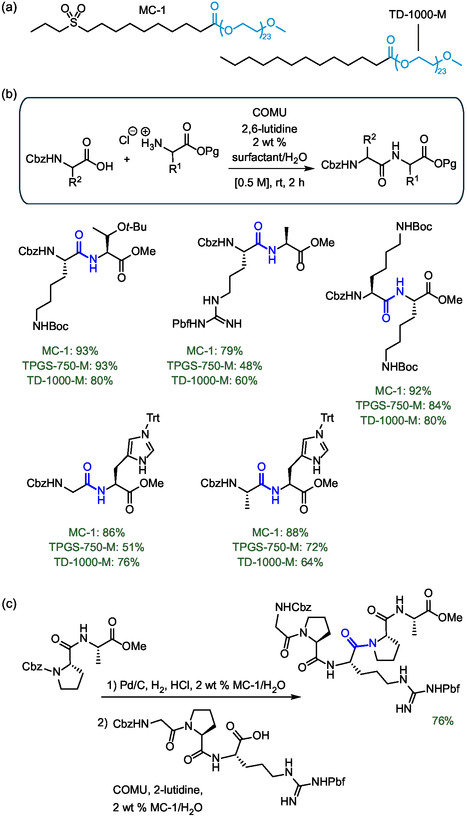
a) Molecular structure of the designer surfactant MC‐1 and TD‐1000‐M. b) Application of MC‐1 in peptide synthesis in water outperforming other surfactants like TPGS‐750‐M. c) Examples of gram‐scale syntheses of the pentapeptide Cbz‐Gly‐Pro‐Arg(Pbf)‐Pro‐Ala‐OMe in water mediated by MC‐1. The chemical moiety that confers water solubility is highlighted in cyan. The peptide bond formed is highlighted in blue.

To advance the application of micellar media for peptide synthesis, Lipshutz and colleagues proposed a new surfactant design named MC‐1 that includes polar side chains. The evolution toward more polar surfactants was driven by the need to overcome previous difficulties encountered in peptide synthesis in the presence of TPGS‐750‐M that often resulted in pasty mixtures that were difficult to mix. The surfactant MC‐1 is characterized by the presence of a rather long hydrophilic PEG 1000 chain connected to a hydrophobic aliphatic chain bearing a sulfone moiety in close proximity to the terminal carbon atom (**Figure** **13**a).^[^ ^60^ ^]^ The similarity of this functional group to the well‐known polar aprotic solvent for peptide synthesis DMSO has provided strong improvements in yields for a wide range of peptides of different lengths. MC‐1 consistently outperformed TPGS‐750‐M in a wide range of 1 pot 2 step deprotection/coupling reactions (Figure 13b). Furthermore, MC‐1 showed much better properties than the similar surfactant TD‐1000‐M lacking the sulfonic moiety, thus confirming its fundamental role (Figure 13a). The robustness of the methodology was demonstrated by replicating several gram‐scale syntheses with essentially unchanged performance. This also includes the synthesis of the pentapeptide Cbz‐Gly‐Pro‐Arg(Pbf)‐Pro‐Ala‐OMe with “botox‐like” anti‐wrinkle properties via a 3 + 2 deprotection/coupling reaction with an overall yield of 76% (Figure 13c).

### 4.3 Surfactant PS‐750‐M

**Figure 14:**
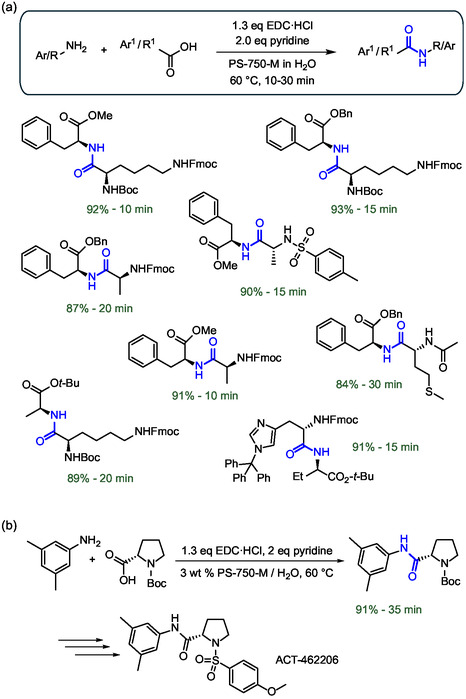
a) Peptide bond formation in water using EDC·HCl as coupling agent and the designer surfactant PS‐750‐M. b) Upscale of the amide bond formation to the multigram for the synthesis of drug intermediate of ACT‐462 206 as a potential candidate for the treatment of insomnia. The peptide bond formed is highlighted in blue.

In terms of reduced environmental impact, low waste production and excellent efficiency, the peptide coupling method proposed by Handa and colleagues is characterized by a wide range of important advantages. This approach relies on the use of the surfactant PS‐750‐M, an amphiphile molecule characterized by the presence of a proline (Pro) linker between the hydrophobic aliphatic side chain and the PEG hydrophilic portion (Figure 9). Interestingly, amide units resembling the polar aprotic solvents dimethylformamide, dimethylacetamide, and *N*‐methyl‐2‐pyrrolidone appear to be critical in promoting efficient peptide bond formation. Once in water, PS‐750‐M provides micelles that promote the solubilization of amino acids which, in combination with 1‐ethyl‐3‐(3‐(dimethylamino)propyl)carbodiimide hydrochloride (EDC·HCl) as a coupling agent, leads to the formation of the corresponding peptides with reaction times of less than 1 h. The rapid activation of the carboxylic acid and the coupling with the amide are important results of this synthetic method, which allowed to avoid the use of hydroxybenzotriazole (HOBt). This latter agent is commonly added to accelerate the coupling and minimize the possible epimerization of the product due to the racemization of the amino acids. The authors applied the coupling method to a wide range of peptides observing good compatibility with *N*‐Boc, *N*‐Cbz, and *N*‐Fmoc protected amino acids, as well as *C*‐protected amino acids with methyl, alkyl, and benzyl ester units (**Figure** **14**a).^[^ ^61^ ^]^ Among the advantages of the PS‐750‐M coupling method in water, it is worth mentioning the possibility of isolating the product simply by filtration and the consequent limited use of solvents during the purification phases.^[^ ^62^ ^]^ This led to average process mass intensity values (PMI, the ratio of the mass of all chemicals used, including solvents, divided by the amount of product obtained) between 18 and 27, values also acceptable for possible upscaling. Further investigations conducted by the authors allowed to ascertain the role of pyridine as a peculiar base of proper strength to ensure that the EDC coupling agent remains protonated and amphiphilic to better interact with PS‐750‐M micelles.^[^ ^63^ ^]^ The authors have in fact applied the system to gram‐scale synthesis, observing substantially unaltered yields and purities of the products through simple filtration, without requiring column chromatography or crystallization (Figure 14b). The commercial availability of PS‐750‐M from major chemical suppliers will certainly spur further applications in peptide synthesis.

### 4.4 Surfactant Savie

**Figure 15:**
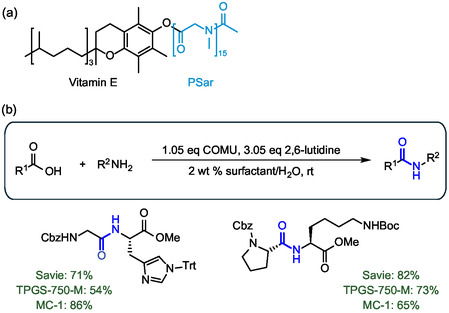
a) Molecular structure of the new designer surfactant Savie for efficient peptide synthesis in water, leading to fully biodegradable water‐based waste. b) Examples of peptides synthesized in water and comparison with other designer surfactants TPGS‐750‐M and MC‐1. The chemical moiety that confers water solubility is highlighted in cyan. The peptide bond formed is highlighted in blue.

Recently the portfolio of designer surfactants was further enriched by the introduction of Savie, a biodegradable tocopheryl polysarcosinate surfactant derived from vitamin E and polysarcosine (**Figure** **15**).^[^ ^64^ ^]^ The key feature of this surfactant is the presence of a hydrophilic poly‐peptide side chain in place of the common PEG unit, which leads to complete biodegradation while minimizing downstream wastewater treatment. Lipshutz and colleagues synthesized Savie using an efficient three‐step one‐pot process from vitamin E and sarcosine *N*‐carboxyanhydride to yield the surfactant as a crystalline solid. Notably, Savie exhibited a four‐fold lower E factor than the reference surfactant TPGS‐750‐M, underscoring the improved environmental impact of this newly designed surfactant. Savie led to enhanced reaction rates and product yields for a wide range of reactions, including peptide synthesis in water (Figure 15). It is worth noting that Savie has enabled better yields than other high‐performance surfactants in water such as TPGS‐750‐M and MC‐1. Furthermore, its “DMF‐like” polyamide side chain promotes effective emulsions compared to other surfactants, leading to better isolated yields. All these exquisite properties have made Savie the surfactant of choice for the development of further peptide syntheses in water.

### 4.5 Additive HPMC

**Figure 16:**
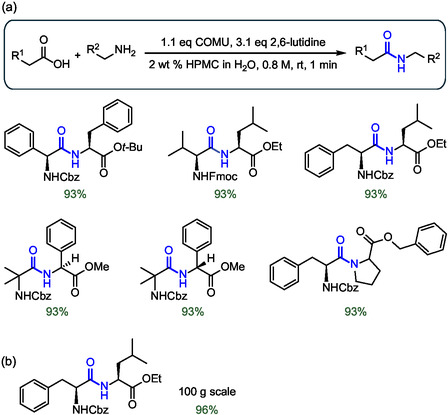
a) Examples of extremely rapid (< 1 min) peptide couplings in water mediated by HPMC. b) Large scale application for the synthesis of a dipeptide mediated by HPMC. The peptide bond formed is highlighted in blue.

To approach peptide synthesis from a process chemistry perspective, Handa and colleagues, in collaboration with the company AbbVie, proposed the use of HPMC as an additive in water (Figure 9).^[^ ^65^ ^]^ HPMC is a natural derivative of polymeric cellulose and exhibits excellent biodegradability and water solubility. For these properties, HPMC is an inactive pharmaceutical ingredient approved by the FDA for a wide range of applications. For example, HPMC finds application as a polymer solubilizer to increase the viscosity of an aqueous medium. By using its alkyl ether side chains and cyclic ether rings, HPMC can effectively provide site‐specific hydrophobic pockets that allow efficient solubilization of the protected amino acid in water, ultimately facilitating very rapid formation of amide bonds with reaction times ranging from seconds to minutes, a great advantage in terms of potential large‐scale applications (**Figure** **16**a).^[^ ^66^ ^]^ The addition of HPMC facilitated the synthesis of several dipeptides with high isolated yields even when non‐natural amino acids such as 2‐aminoisobutyric acid were used. An efficient scale‐up was demonstrated in the synthesis of 100 g of the dipeptide Cbz‐Phe‐Val‐OEt, also achieved in a reaction time of less than 1 min (Figure 16b). To be used efficiently in water, HPMC requires gentle heating of the reaction mixture to 45–60 °C. Such high temperatures promote its reversible gelation leading to the formation of hydrophobic pockets that enable organic transformations. Therefore, crucial temperature optimization and proper selection of the HPMC grade are essential for the success of this aqueous technology.

### 4.6 Other Additives

**Figure 17:**
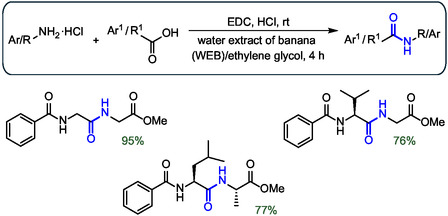
Examples of peptide synthesis in WEB that do not require the presence of a base. The peptide bond formed is highlighted in blue.

To further highlight the feasibility of peptide formation in water and its combination with waste valorization, it is interesting to mention that peptide bond formation could also be performed in aqueous banana extract (WEB). The WEB extract is obtained by drying banana peels, then burning them to ash, mixing them with water, and finally filtering them. This aqueous environment worked much better than pure water for a wide range of combinations of amino acids not bearing protected side chains using EDC as the coupling agent (**Figure** **17**).^[^ ^67^ ^]^ The natural presence of sodium and potassium carbonates in banana peels, which can act as bases, not only avoided the addition of exogenous bases but also favored the use of non‐Fmoc‐protected amino acids.

## 5 Summary and Outlook

Water, nature's preferred reaction medium for biological reactions, is also gaining attention for chemical peptide synthesis. This in turn is driven by increasing environmental awareness and the demand for minimizing waste, especially for organic solvents. However, the state‐of‐the‐art of peptide synthesis in aqueous media is not yet mature enough to be dominated by a single method. Rather, there are some notable successful approaches, classified by the use of water‐soluble solid supports or solution‐based synthesis that are considered the best existing strategies to date. For SPPS, the simplest approaches directly use standard Fmoc‐ or Boc‐protected amino acids as nanoparticles with water‐soluble resins and coupling agents. Alternatively, for LPPS, the use of surfactants or additives offers great advantages in terms of reaction speed, minimized epimerization, easy product isolation, and, very importantly, possible scale‐up. In all cases, a large minimization of organic solvents, especially polar aprotic ones, is observed, which is the spurring idea behind this new field of research. Further improvements are possible by combining peptide synthesis in water with microwave irradiation, though with precise control and determination of benefits, as this technique is often overestimated in terms of efficiency.^[^ ^68^ ^]^

A deeper understanding of the key requirements for effective peptide synthesis in aqueous media could also help improve our understanding of biological peptide synthesis, aiding possibly better mimic of biosynthetic pathways. In this direction, a recent publication has demonstrated the possible synthesis of peptides in water through the use of dipyridyldithiocarbonate (DPDTC) *via* the formation of thioesters as activated species, similar for example to what occur in non‐ribosomal peptide synthetases.^[^ ^69^ ^]^ Similar to what has been observed for many other benchmarks, the use of water as an alternative media in peptide synthesis requires a critical dissemination of known methods, to stimulate further other groups to explore this fascinating topic.

## Conflict of Interests

The authors declare no conflict of interest.

## Author Contributions

**Alessandro Angelini**: conceptualization (equal); funding acquisition (lead); supervision (equal); writing—original draft (equal); writing—review and editing (equal). **Alessandro Scarso**: conceptualization (equal); data curation (equal); supervision (equal); writing—original draft (equal); writing—review and editing (equal). **Francesca Bordignon**: data curation (equal); writing—original draft (equal); writing—review and editing (equal).
